# Chronic restraint stress induces sperm acrosome reaction and changes in testicular tyrosine phosphorylated proteins in rats

**Published:** 2016-07

**Authors:** Supatcharee Arun, Jaturon Burawat, Wannisa Sukhorum, Apichakan Sampannang, Chanwit Maneenin, Sitthichai Iamsaard

**Affiliations:** 1 *Department of Anatomy, Faculty of Medicine, Khon Kaen University, Khon Kaen, Thailand.*; 2 *Center for Research and Development of Herbal Health Products, Faculty of Pharmaceutical Sciences, Khon Kaen University, Khon Kaen 40002, Thailand.*

**Keywords:** *Stress*, *Sperm*, *Acrosome*, *Testis*, *Phosphorylation*

## Abstract

**Background:**

Stress is a cause of male infertility. Although sex hormones and sperm quality have been shown to be low in stress, sperm physiology and testicular functional proteins, such as phosphotyrosine proteins, have not been documented.

**Objective:**

To investigate the acrosome status and alterations of testicular proteins involved in spermatogenesis and testosterone synthesis in chronic stress in rats.

**Materials and Methods:**

In this experimental study, male rats were divided into 2 groups (control and chronic stress (CS), n=7). CS rats were immobilized (4 hr/day) for 42 consecutive days. The blood glucose level (BGL), corticosterone, testosterone, acrosome status, and histopathology were examined. The expressions of testicular steroidogenic acute regulatory (StAR), cytochrome P450 side chain cleavage (CYP11A1), and phosphorylated proteins were analyzed.

**Results:**

Results showed that BGL (71.25±2.22 vs. 95.60±3.36 mg/dl), corticosterone level (24.33±4.23 vs. 36.9±2.01 ng/ml), acrosome reacted sperm (3.25±1.55 vs. 17.71±5.03%), and sperm head abnormality (3.29±0.71 vs. 6.21±1.18%) were significantly higher in CS group in comparison with control. In contrast, seminal vesicle (0.41±0.05 vs. 0.24±0.07 g/100g), testosterone level (3.37±0.79 vs. 0.61±0.29 ng/ml), and sperm concentration (115.33±7.70 vs. 79.13±3.65×10^6^ cells/ml) of CS were significantly lower (p<0.05) than controls. Some atrophic seminiferous tubules and low sperm mass were apparent in CS rats. The expression of CYP11A1 except StAR protein was markedly decreased in CS rats. In contrast, a 55 kDa phosphorylated protein was higher in CS testes.

**Conclusion:**

CS decreased the expression of CYP11A, resulting in decreased testosterone, and increased acrosome-reacted sperm, assumed to be the result of an increase of 55 kDa phosphorylated protein.

## Introduction

Chronic stress is a condition that is induced by any stressor for a long-term period. It is known that stress affects the physiological functions of the central nervous, cardiovascular, digestive, and neuroendocrine systems, as well as the reproductive system. Biochemical analysis has shown increased corticosterone and blood glucose levels to be major markers of stress conditions (1-3). Many reports have demonstrated that psychological and physiological stress could induce sexual dysfunction and infertility (4, 5). In males, stress significantly decreases the levels of gonadotropin-releasing hormone, follicle stimulating hormone (FSH), luteinizing hormone (LH), and testosterone (3, 4, 6). Such effects might impair sperm quality (3, 7-8). 

Previous studies have reported that stress also induces Leydig cell and seminiferous tubule damages (3, 9, 10). Many reports explain the testosterone production decline in conditions of acute stress events and demonstrated that expressions of steroidogenic proteins and enzymes such as steroidogenic acute regulatory (StAR) protein and cytochrome P450 side chain cleavage (CYP11A1) enzyme to be low (2, 11, 12). However, changes of sperm physiology and other testicular markers in conditions of stress require further explanation. Sperm acrosome reaction is a specialized exocytotic event of fusion and fenestration between sperm plasma and the outer acrosomal membrane (13-16). After sperm capacitation, this physiological process results the extreme release of various hydrolytic enzymes to digest the zona pellucida (ZP) in facilitation of successful sperm-ZP penetration in early fertilization process. Premature or precocious acrosome reaction can be a cause of male infertility. There are some substances that shown the increase in percentage of acrosome-reacted sperm (17-19). 

Moreover, tyrosine phosphorylations are important in spermatogenesis, capacitation, and acrosome reaction (13, 14, 20). Phosphorylated proteins have been shown to play a role in spermiogenesis in testes, since they are localized only in Sertoli cells and elongated spermatids (21). The patterns of testicular phosphorylated proteins have been shown in other treatments (3, 18, 22). In previous literatures, the adverse mechanisms of male reproductive effects underlying chronic restraint stress especially acrosome status and expression of testicular phosphorylated protein in animals have never been demonstrated. 

In addition to demonstrate the adverse male reproductive parameters, this study attempted to investigate the epididymal sperm acrosome status and changes of testicular phosphorylated proteins in rats suffering from chronic stress.

## Materials and methods

**Animals and chronic stress exposure**

Fourteen male Sprague-Dawley rats (100-150 gr) were purchased from the National Laboratory Animal Center, Mahidol University, Salaya, Nakhon Pathom, Thailand. This experiment was approved by the Animal Ethics Committee of Khon Kaen University, based on the Ethics of Animal Experimentation as determined by the National Research Council of Animal Experimentation. 

Animals were housed in stainless cages under controlled environmental conditions (temperature 22±2^o^C, 12 hr light/dark cycles, humidity 30-60% RH, sound <85 decibels, light intensity 350-400 lux). They received daily commercial pellet food and water *ad libitum. *Animals were divided into two groups (n=7). 

Rats in the control group were not exposed to the stressor, while rats in the chronic stress (CS) group were immobilized using a restraint cage for 4 hr/day for 42 consecutive days to induce chronic stress as described in previous studies (6, 9). Body weight was measured daily. 

**Blood glucose levels and plasma hormone analysis**

The blood glucose levels were measured by pricking the tip of rats’ tails with test strips and using a blood glucose oxidase reaction monitoring system after fasting (Johnson and Johnson Ltd., USA) (23). At the end of experiment, all rats were euthanized by cervical dislocation and blood was collected from the heart by cardiac puncture and centrifuged at 13,000 rpm at 4^o^C for 7 min using a micro centrifuge (Beckman Coulter^TM^, USA) to separate the plasma from the blood cells. After that, the levels of plasma corticosterone and testosterone were measured by radioimmunoassay at the Radiology Unit, Srinagarind Hospital, Faculty of Medicine, Khon Kaen University, Thailand.

**Morphological and histological studies of reproductive organs**

After euthanasia, male reproductive organs (testis, epididymis plus vas deferens, seminal vesicle, and penis) were collected and weighted. The absolute weights of the reproductive organs were calculated and expressed as relative weight (g/100g). Subsequently, the gross structures of these organs were observed and photographed using a digital camera (Nikon Coolpix S2600, Japan). For histological examination, right testis and caudal epididymis were fixed in 10% formalin for 2 days (pH=7.4). These tissues were then routinely processed for light microscope technique. After that paraffinized-tissue blocks were sectioned at 7 µm and stained with hematoxylin and eosin (H&E). The histological images were photographed using a Nikon light ECLIPSE E200 microscope equipped with a DXM1200 digital camera.

**Sperm count and head morphology examination**

The left caudal epididymis and vas deferens were gently operated and squeezed to collect sperm fluid. Following this, epididymal sperm fluid was dipped and suspended in 1 ml of phosphate buffer saline (PBS, 37^o^C, pH=7.4). Subsequently, the diluted sperm suspension was centrifuged at 5,000 rpm at 25^o^C for 2 min to wash and separate the mature sperm pellet from its fluid. The sperm pellet was re-suspended with 1 ml PBS. The sperm suspension was subsequently diluted with PBS (1:20 dilution) before counting. The diluted sperm suspension (10 µl) was dropped on the Neubauer counting chamber. After that, the sperm was counted under a light microscope (Nikon ECLIPSE E200, Japan) in triplicate examinations (18). 

To examine abnormal sperm heads, the diluted sperm was smeared on glass slide and dried in a hot-air oven at 50^o^C overnight (24, 25). The dried sperm was then fixed with methyl alcohol for 20 min and stained using hematoxylin (20 min) and eosin (5 min). After washing, the stained slide was dehydrated with an ethanol series for 5 min. After observation, types of abnormal sperm heads were classified as has been described in previous study (25). Representative sperm heads were then photographed using a Nikon light ECLIPSE E200 microscope equipped with a DXM1200 digital camera. In each animal, two hundred sperm cells were counted and used to calculate the percentage of sperm-head abnormality in the total number.

**Sperm acrosome reaction assay**

To determine the sperm acrosome reaction, the diluted-sperm suspension was gently smeared on a gelatin-coated slide. Then, the sperm was dried at room temperature for 24 hr. The dried sperm was subsequently stained with 0.22% Coomassie blue for 5 min. After washing, the stained-sperm slide was mounted using mounting media. Sperm acrosomes were classified into two categories (18, 26). Sperm presenting an acrosome cap stained with Coomassie blue was classified as acrosome intact (AI) sperm, whereas sperm without staining on acrosome cap was classified as acrosome reacted (AR) sperm. 

Representative acrosome status (AI or AR) was subsequently photographed by a Nikon light ECLIPSE E200 microscope equipped with a DXM1200 digital camera. Finally, two hundred total sperm cells were counted and the percentage of acrosome reacted status was calculated.

**Immuno-Western blotting analysis of StAR, CYP11A1, and tyrosine phosphorylated protein expressions**

To extract testicular proteins, the testis was homogenized with RIPA buffer (Cell Signaling Technology, Inc., USA) containing a cocktail of protease inhibitors (Sigma, Inc., USA). Then, the homogenized-testicular lysate was centrifuged at 12,000 rpm at 4^o^C for 10 min to separate testicular soluble proteins from the pellet. Total protein concentration of testicular lysate was measured using a NanoDrop spectrophotometer (ND-1000 NanoDrop Technologies, Inc., USA) at an absorbance of 280 nm. 

To determine expressions of testicular StAR, CYP11A, and phosphotyrosine proteins, all of the proteins (80 µg) were loaded and separated in 10% sodium dodecyl sulfate polyacrylamide gel (SDS-PAGE). Separated proteins on SDS gel were transferred onto the nitrocellulose membrane. Then, all membranes were incubated with 5% skim milk in 0.1% PBST (0.1% Tween-20, PBS, pH=7.4) for 1 hr to block non-specific binding proteins and individually incubated with StAR (1:1000 dilution; Santa Cruz Biotechnology, Inc., USA), CYP11A1 (1:1000 dilution; Santa Cruz Biotechnology, Inc., USA), or β-actin antibody (1:2000 dilution; Santa Cruz Biotechnology, Inc., USA) at 4^o^C overnight.

Then, the membrane was washed in 0.05% PBST (0.05% Tween-20, PBS, pH=7.4) for 5 min (3 times) and incubated with specific secondary antibody conjugated with horseradish peroxidase goat anti-rabbit IgG (1:2000 dilution) for anti-StAR, donkey anti-goat IgG (1:2000 dilution) for anti-CYP11A1, or goat anti-mouse IgG (1:2000 dilution) for anti β-actin) for 1 hr at room temperature. In order to analyze tyrosine phosphorylated protein expression, the transferred-protein membrane was incubated with anti-phosphotyrosine primary antibody (1:2000; Millipore Co., USA) at 4^o^C overnight. It was then washed and incubated with anti-mouse secondary antibody for 2 hr at room temperature (19). 

All membranes were washed with 0.05% PBST for 5 min (3 times) before detections of those marker proteins by using enhanced chemiluminescence (ECL) substrate under a gel doct apparatus (ImageQuant 400, GH Healthcare, USA). To reveal the actual reactivity of individual primary antibodies, the StAR lysate, EGF stimulated A413 cell lysate or β-actin was used as positive control. 

**Statistical analysis**

All quantitative data were represented as mean±SD. The independent sample* t*-test was performed to examine the significant differences between groups using SPSS statistics 19.0 software (Statistical Package for the Social Sciences, version 19.0, SPSS Inc, Armonk, New York, USA). P<0.05 was considered significant difference.

## Results

**Effect of CS on corticosterone and blood glucose levels**

**Figure 1:**
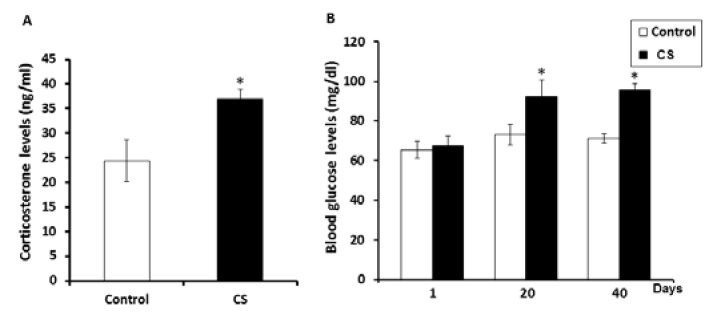
Showing the corticosterone (A) and blood glucose levels (B) of the control and chronic stress (CS) groups. Data are represented as mean ± S.D. (n=7 rats each group). *p<0.05

The corticosterone levels (Figure 1A) and blood glucose levels (Figure 1B) at days 20 and 40 of the chronic stress group were significantly increased (p<0.05) as compared to control group.

**Effect of CS on body weight and male reproductive organs**

**Figure 2:**
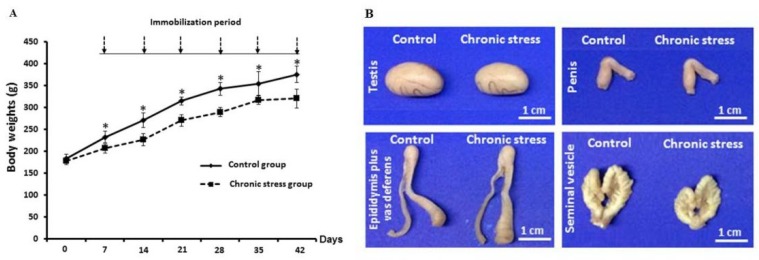
Comparison of the changes in body weight each week during experiment (A) and the morphological photographs of testis, penis, epididymis plus vas deferens, and seminal vesicle (B) between the control and chronic stress groups. Each data point represented as mean ± S.D. (n=7 rats each group). *p<0.05

The body weight of CS group had decreased significantly (p<0.05) each week as compared to the control group (Figure 2A). However, it was observed that the morphologies of testis, penis, epididymis plus vas deferens did not vary between groups. In contrast, the seminal vesicle of CS group was observably smaller than control (Figure 2B). The size (width×length) of seminal vesicle in control group was 1.54±0.07×2.46±0.07 cm and CS group was 1.42±0.07×1.58±0.07 cm approximately (Figure 2B). 

**Effect of CS on reproductive organ weight, testosterone hormone, sperm concentration, sperm head abnormality, and acrosome-reacted sperm**

**Figure 3:**
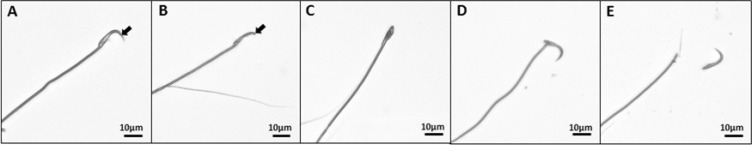
Photographs showing sperm head morphologies (400×) of control and chronic stress groups. Normal sperm head with hook (A). Abnormal sperm heads; without hook (B), pinhead (C), crooked neck (D), and tailless head (E)

The absolute and relative weights of reproductive organs are shown in table I. The absolute weights of testis, epididymis plus vas deferens, and seminal vesicle in CS group were significantly lower in comparison with control. All relative weights except the seminal vesicle were not significantly different between groups. In addition, CS significantly lowered testosterone level and sperm concentration whereas sperm head abnormality had significantly increased as compared with control group (Table I). The sperm head morphologies were shown in figure 3. As compared to the normal sperm (Figure 3A), the abnormal sperm heads consisting of without hook (Figure 3B), pinhead (Figure 3C), crooked neck (Figure 3D), and tailless head (Figure 3E) in CS group had increased significantly (Table I).

**Effect of CS on sperm acrosome status**

**Figure 4:**
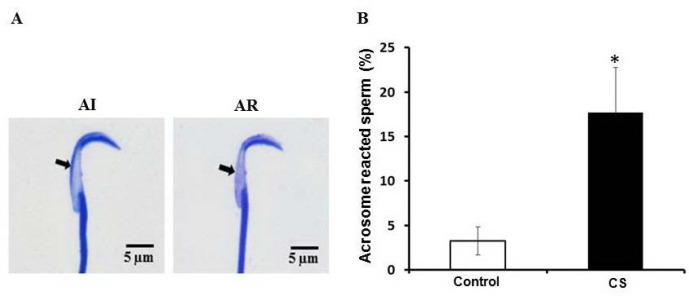
Photographs showing sperm acrosome status (A) stained with Coomassie blue and the percentage of acrosome reacted sperm observed in control and chronic stress groups (B) AI: acrosome intact sperm, AR: acrosome reacted sperm (1000×). Data are represented as mean±SD. (n=7 rats each group). *p<0.05

Sperm stained with Coomassie blue to acrosome status is shown in figure 4A. After quantitative analysis, the percentage of acrosome-reacted sperm of CS had significantly increased (p<0.05) as compared with control (Figure 4B). 

**Effect of CS on testis histology and caudal epididymis**

**Figure 5:**
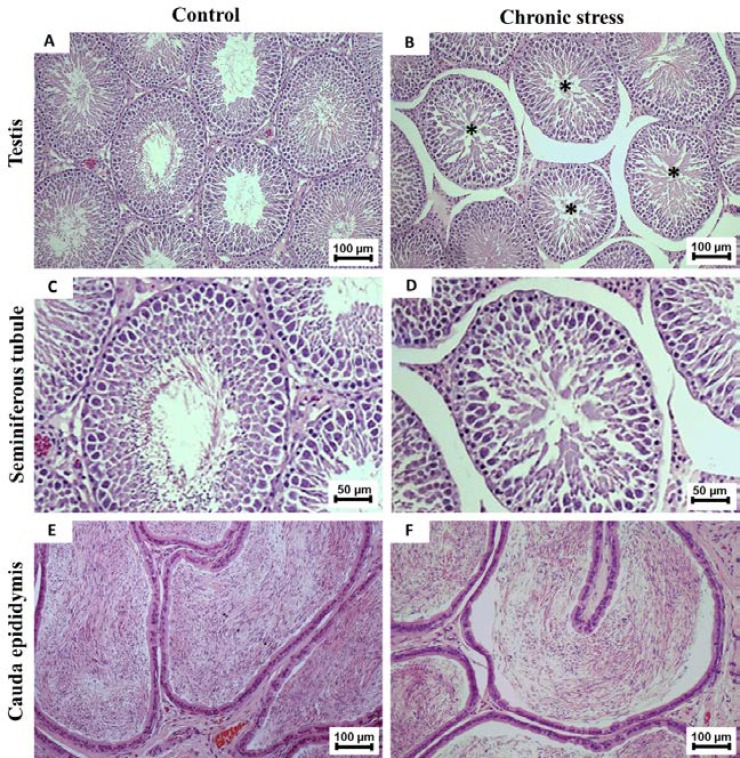
Histology (H&E) of testis (A and B), seminiferous tubule (C and D), and caudal epididymis (E and F) compared between control and chronic stress groups. Asterisks: seminiferous tubular atrophy with disorganization of germ cells. *Note*: widespread interstitial spaces were obviously found in CS group (B and D). (A and B 100×; C and D 400×; E and F; 100×)

The testicular and caudal epididymis histology was shown in figure 5*.* These results demonstrated that CS induced an increase of seminiferous tubular atrophy and interstitial space as compared with control (Figure 5A-5D). In addition, the CS group was found to have a lower density of caudal sperm than the control group (Figure 5E, 5F), which associated with a decrease in sperm concentration (Table I).

**Effect of CS on expressions of StAR and CYP11A1 proteins**

**Figure 6:**
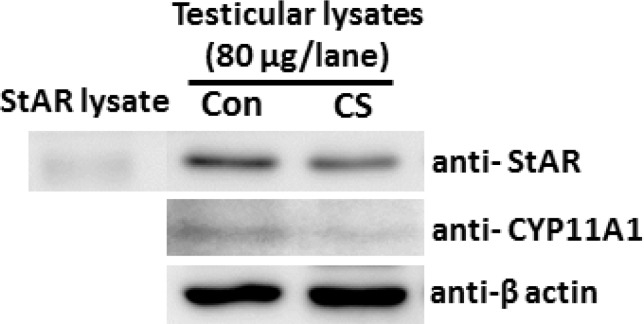
Representative immuno-Western blot of testicular StAR and CYP11A1 proteins compared between control and chronic stress groups. The StAR lysate was used as a positive control for anti-StAR. β -actin was used as internal control for all antibodies.

As shown in figure 6, the CS did not affect the expression of testicular StAR protein as compared to control. In contrast, the expression of testicular CYP11A1 protein had observably decreased compared to control group (Figure 6). Equally-loaded proteins of both groups were confirmed by equal β-actin (Figure 6). 

**Effect of CS on expression of tyrosine phosphorylated protein**

**Figure 7:**
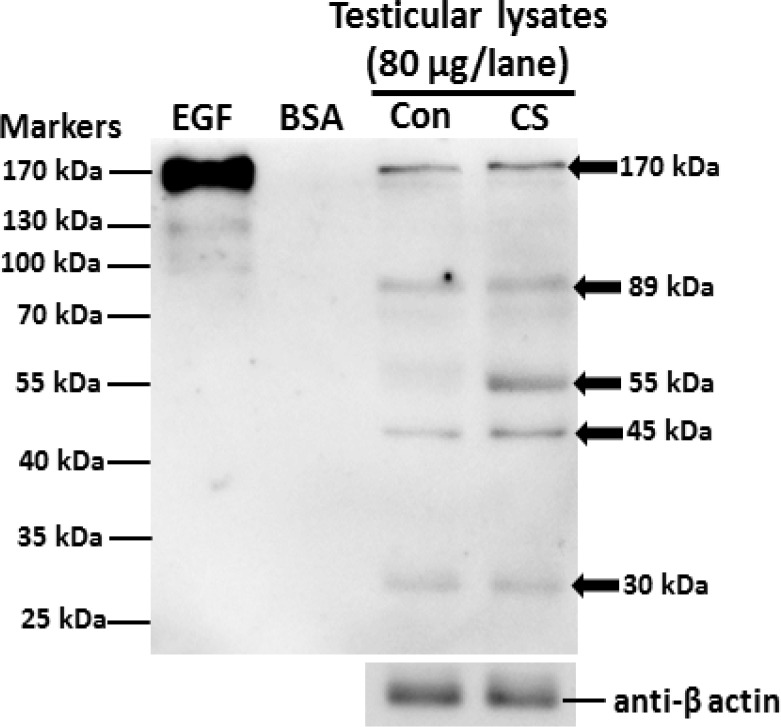
Representative immuno-Western blot analysis of tyrosine phosphorylated protein expression in testis of control and chronic stress groups. Epidermal growth factor (EGF)-like factor and bovine serum albumin (BSA) were used as positive and negative controls respectively. β -actin was used as an internal control.

The expression patterns of tyrosine phosphorylated proteins are shown in figure 7. The result shows five major bands (30, 45, 55, 89, and 170 kDas respectively) of testicular tyrosine phosphorylated proteins in both groups. 

Remarkably, the expression of a phosphorylated 55 kDa protein had increased in CS group as compared to the control (Figure 7).

**Table I:** Effect of chronic stress on male reproductive organ weights, testosterone level, sperm concentration, and sperm head abnormality

**Parameters**		**Control**	**Chronic stress**
Testis			
	Absolute weight (g)	1.91 ± 0.10	1.78 ± 0.08^*^
	Relative weight (g/100g)	0.55 ± 0.01	0.54 ± 0.01
Epididymis plus vas deferens			
	Absolute weight (g)	0.70 ± 0.04	0.65 ± 0.02^*^
	Relative weight (g/100g)	0.20 ± 0.01	0.18 ± 0.00
Seminal vesicle			
	Absolute weights (g)	1.52 ± 0.11	1.14 ± 0.10^*^
	Relative weight (g/100g)	0.41 ± 0.05	0.24 ± 0.07^*^
Testosterone level (ng/ml)		3.37 ± 0.79	0.61 ± 0.29^*^
Sperm concentration (10^6^cells/ml)		115.33 ± 7.70	79.13 ± 3.65^*^
Sperm head abnormality (%)		3.29 ± 0.71	6.21 ± 1.18^*^

* Significant differences (p<0.05). Data are represented as mean ± S.D. (n=7 rats each group).

## Discussion

Previous studies have demonstrated that stress disturbs the normal homeostasis on the various body systems (27). In males, stress is also a major cause of infertility resulting from factors such as decreased sex hormones, erectile dysfunction, delayed ejaculation, orgasmic difficulty, low sexual desire, and low sperm quality (4, 5). Consistent with other studies, this study showed that CS significantly increases corticosterone and blood glucose levels while decreasing testosterone levels (1, 6, 23, 28-30). 

In general, stress stimulates the increase of corticosterone via the hypothalamic-pituitary-adrenal (HPA) axis (30). Consequently, increased corticosterone can stimulate gluconeogenesis in liver and muscle tissues resulting in elevated blood glucose levels (23). In addition, corticosterone stimulation has shown that decreased testosterone levels in Leydig cells resulted from lower expressions of scarvenger receptor class B (Scarb1), steroidogenic acute regulatory (StAR) protein, cytochrome P450 side chain cleavage (CYP11A1), cytochrome P450 17α-hydroxylase (CYP17A1), and 17alpha-hydroxysteroid dehydrogenase (Hsd17b3) enzymes (2, 11, 12, 31). 

This study showed a significant decrease of body weight and seminal-vesicle size in CS group. It is possible that the high levels of corticosterone in stressed rats stimulated protein catabolism and lipolysis, whereas lower protein synthesis was resulted from decreased testosterone levels. Indeed, this is the first study to show low expression of testicular CYP11A1 but not StAR protein in stressed animals, supporting a significant decrease of plasma testosterone level in CS group. In addition, the low sperm quality was observed in stressed rats. The decline of sperm concentration in rats suffering from CS might be the consequence of significant decrease in testosterone, leading to spermatogenesis. This was confirmed by the presence of seminiferous tubule atrophy and low sperm mass in epididymis of CS rats, similarly observed in previous investigations (3, 10, 32). 

Additionally, the abnormal head and premature acrosome-reacted sperm in epididymis had increased significantly in CS group, which was similar to the acute immobilization stress model previously reported by Arun and co-workers. In differences to the acute restraint stress for 7 days, the present study showed that chronic restraint stress (42 days) could increase the tailless sperm head type, exocytosed acrosome, a testicular 55 kDa phosphorylated protein (3). 

In addition, the expression of testicular CYP11A1 in CS was obviously decreased. Such evidences have never been investigated in acute stress model but they only found the decreased expression of a testicular 95 kDa phosphorylated protein (3). We assumed that CS particularly affected the formation process of sperm heads, also called “spermiogenesis”. Previous studies have shown that some substances can increase sperm acrosome reaction in rats (17-19). 

It is possible that stress might affect the regulation of some proteins involved in acrosome formation such as Golgi-associated PDZ- and coiled-coil motif-containing (GOPC), and autophagy-related 7 (Atg7) proteins (33-34). The protein phosphorylation is essential for cell proliferation, division, growth, and differentiation. This process is also found in Sertoli cells and round spermatids of testes and in acrosome reaction process (21). 

Therefore, it is assumed to be involved in sperm acrosome formation at spermiogenesis. Interestingly, the expression of a testicular 55 kDa phosphorylated protein in rats exposed to CS had observably increased. This protein may be important for spermatogenesis especially in sperm-head and acrosome formations. However, it is necessary to investigate the actual structure and function of this protein further. 

## Conclusion

In conclusion, the chronic restraint stress increased the percentages of sperm acrosome reaction and head abnormalities in a rat model. It could decrease the expression of testicular CYP11A1 protein resulting in significant reduction of serum testosterone levels and sperm production. Although the overexpression of a testicular 55 kDa phosphorylated protein in stress rats could not be clearly explained. 

We suggested that this protein might involve in suppressions of spermatogenesis or acrosome forming because phosphorylated proteins have been localized in Sertoli cells and spermatids. 

## Note

***This article extracted from Ph.D. thesis. (Supatcharee Arun)***
